# Randomised primary health center based interventions to improve the diagnosis and treatment of undifferentiated fever and dengue in Vietnam

**DOI:** 10.1186/1472-6963-10-275

**Published:** 2010-09-21

**Authors:** Hoang L Phuong, Tran TT Nga, Phan T Giao, Le Q Hung, Tran Q Binh, Nguyen V Nam, Nico Nagelkerke, Peter J de Vries

**Affiliations:** 1 Division of Infectious Diseases, Tropical Medicine and AIDS, Academic Medical Center, Amsterdam, the Netherlands; 2 Department of Tropical Diseases, Cho Ray Hospital, Ho Chi Minh City, Vietnam; 3 Department of Microbiology, Cho Ray Hospital, Ho Chi Minh City, Vietnam; 4 Binh Thuan Medical College, Phan Thiet, Binh Thuan, Vietnam; 5 Department of Community Medicine, United Arab Emirates University, Al Ain, United Arab Emirates

## Abstract

**Background:**

Fever is a common reason for attending primary health facilities in Vietnam. Response of health care providers to patients with fever commonly consists of making a presumptive diagnosis and proposing corresponding treatment. In Vietnam, where malaria was brought under control, viral infections, notably dengue, are the main causes of undifferentiated fever but they are often misdiagnosed and inappropriately treated with antibiotics.

This study investigate if educating primary health center (PHC) staff or introducing rapid diagnostic tests (RDTs) improve diagnostic resolution and accuracy for acute undifferentiated fever (AUF) and reduce prescription of antibiotics and costs for patients.

**Methods:**

In a PHC randomized intervention study in southern Vietnam, the presumptive diagnoses for AUF patients were recorded and confirmed by serology on paired (acute and convalescence) sera. After one year, PHCs were randomized to four intervention arms: training on infectious diseases (A), the provision of RDTs (B), the combination (AB) and control (C). The intervention lasted from 2002 until 2006.

**Results:**

The frequency of the non-etiologic diagnosis "undifferentiated fever" decreased in group AB, and - with some delay- also in group B. The diagnosis "dengue" increased in group AB, but only temporarily, although dengue was the most common cause of fever. A correct diagnosis for dengue initially increased in groups AB and B but only for AB this was sustained. Antibiotics prescriptions increased in group C. During intervention it initially declined in AB with a tendency to increase afterwards; in B it gradually declined. There was a substantial increase of patients' costs in B.

**Conclusions:**

The introduction of RDTs for infectious diseases such as dengue, through free market principles, does improve the quality of the diagnosis and decreases the prescription of antibiotics at the PHC level. However, the effect is more sustainable in combination with training; without it RDTs lead to an excess of costs.

## Background

Fever is a common reason for attending primary health facilities in developing countries. Recently, we showed that fever caused over 12% of consultations at community primary health facilities in southern Vietnam, next to diarrhoea, respiratory tract infections and preventive measures such as vaccinations [1]. Dengue was the main cause of undifferentiated fevers. Malaria was brought under control during the nineteen nineties [2]. Because of its often non-specific presentation dengue is frequently misdiagnosed and inappropriately treated with antibiotics [3].

The response of health care providers to patients with fever commonly consists of making a presumptive diagnosis and proposes a corresponding treatment. Little is known about these processes. Prescription of drugs, especially antibiotics, is common even for fevers of (presumably) viral origin that are probably self-limiting [3-5]. The resolution and accuracy of presumptive diagnoses have rarely been studied under the conditions of routine primary health care in resource poor conditions. Most studies in developing countries tend to be observational exploring the use of antibiotics, self-medication and over the counter purchase, while intervention studies are mainly in-hospital studies in industrialized countries that aim to redress antibiotic resistance by changing treatment policies [6-13]. Intervention studies that aim to improve the diagnosis and treatment elements of syndromic approaches to fever at the primary health care level in developing countries have never been published to our knowledge. Such interventions could aim to improve the knowledge and diagnostic skills of health care providers, or introduce new, simple, diagnostic tests in their routine diagnostic process to confirm presumptive diagnoses. A wealth of aggressively marketed rapid diagnostic tests (RDTs) has become available but whether these assays really improve diagnostic resolution and accuracy and not only add costs has not been studied properly [14]. In contrast to malaria, where improving diagnostic facilities can be very efficient in reducing over diagnosis and over treatment, the approach to fevers other than malaria is much more difficult [15].

In Binh Thuan, southern Vietnam, malaria was brought under control, but its primary health services are still being consulted by large numbers of patients with fever [1,16], who often receive antibiotics without a proper differential diagnosis.

In this study we investigate which interventions, viz. improving knowledge or providing rapid diagnostic tests, effectively improve the diagnostic resolution and accuracy of health care professionals, and whether these effectively reduce antibiotics prescriptions and costs for patients.

## Methods

### Study area

**Figure 1:**
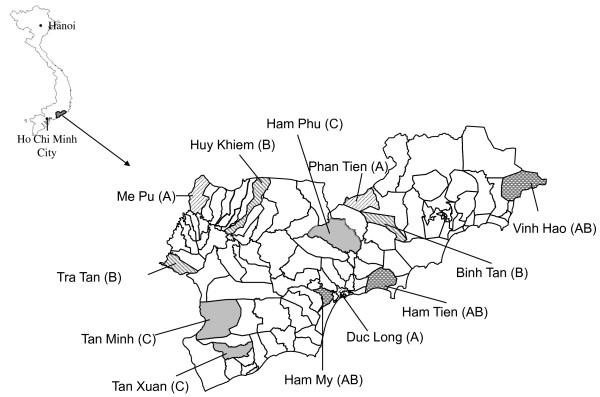
**Map**. Administrative map of Binh Thuan indicating the communes (name and number) participating in the study. Between brackets the intervention group.

A community randomized trial with a pre-intervention period and an intervention period with four study arms [17] was conducted in Binh Thuan province in southern Vietnam, between March 2001 and March 2006, an area that has been described previously [3]. Demographic and socioeconomic indicators are shown in Table 1. Twelve PHCs were selected. The sample size of participating PHCs was based on feasibility and the aim to include communities that were not adjacent to one another and represented the different demographic and ecological zones of the province. (Figure 1) Added to these twelve PHCs was the clinic of the Provincial Malaria Control Center in the capital, Phan Thiet.

**Table 1:** Characteristics of the 12 communities in a randomized primary health centers based intervention study in Binh Thuan Province, Vietnam.

	Binh Thuan Province	PHC intervention groups			
		Training	RDTs	Training + RDTs	Control
**Administrative data**					
No. Communities in 2001	124	3	3	3	3
No. households	-	5725	5428	6457	6064
Population	1089328	29037	29700	33511	28332
Per capita income (US $)	298				
					
Health service data 2001/2006*					
Total no. registered health providers	1897/2452	30/37	17/19	18/17	16/17
Medical doctors (per 10000 population)	483 (4)/579 (5)	8 (3)/9 (3)	4 (1)/4 (1)	3 (1)/4 (2)	2 (1)/4 (2)
Assistant doctors	927/1068	17/17	8/8	13/12	8/7
Nurses/midwifes/technicians	487/805	5/11	5/7	2/1	6/6
No. PHC with FBC capacity	NA	2/2	1/3	1/3	1/1
No. Malaria patients	6600/448	152/34	242/18	177/5	458/8

* the data of the year 2001 and 2006, separetely

FBC: full blood count

NA: Not available

In 2000, 40% of primary health centers (PHCs) in Binh Thuan were staffed with at least one medical doctor (MD). All PHCs had a so called "assistant doctor" for paediatrics and obstetrics who followed a three-year training program in a medical school or a midwife with a two-year training program. Community health workers were active in all communities and hamlets [18].

Circumstances changed during this study, parallel to the national policy to increase the number of PHCs with at least one MD to ≥ 80% in 2010, by both recruiting new doctors and by providing three years of additional academic training to selected already employed assistant doctors [19]. Otherwise, additional professional training after formal graduation is virtually non-existent. Other changes are the development of a private sector and health insurance [20]. In 2002 health insurance covered approximately 16% of the total population [21]. During our study period patients without health insurance had to pay a small fee (1000 VND or ± US$0.06) for consultation at a PHC and paid for costs of additional diagnostics or drugs. Poor patients and those from ethnic minority groups were exempted from payment.

### Participants

Primary health staff who worked in patient consultation at 12 PHCs and the clinic of the Provincial Malaria Control Center participated in this study. They recruited the patients with acute undifferentiated fever (AUF), defined as any febrile illness of duration less than 14 days, confirmed by an axillary temperature ≥ 38.0°C, without any indication of either severe systemic or organ specific disease. However, only doctors who participated throughout the entire study period, the "permanent health staff", and their patients were eligible for analysis of repeated measures over time. Malaria was excluded by thick blood smear microscopy.

The study was approved by the Review Board of the Cho Ray Hospital, the provincial health services of Binh Thuan and the peoples committees of the participating communities. All patients (or, for children, the parents or guardian) gave written informed consent. A team of study monitors from Cho Ray Hospital in Ho Chi Minh City paid monthly visits to all participating centers.

### Serology

All patients were asked to give serum samples for sero-diagnosis during the acute (at first presentation - t0) and convalescence (after 3 weeks - t3) phase. These samples were stored at -20°C at the study sites until monthly transfer to Cho Ray hospital, where they were stored at -70°C.

Serological confirmation was conducted on patients with paired serum samples (acute and convalescent samples), mostly for Dengue virus infection (IgG and IgM-Capture ELISA, Focus Technologies Inc., Cypress, CA, USA) and other infections such as viral respiratory tract infections (combined IgM/IgA capture ELISA; Meddens Diagnostics BV, Vorden the Netherlands; IgG and IgA ELISA Virion\Serion GmbH, Würzburg, Germany); leptospirosis (Leptospira IgG and IgM Virion\Serion; inhouse-ELISA and MAT, the Royal Tropicical Institute - KIT, the Netherlands [22,23]; Cytomegalovirus and Epstein-barr virus (VCA ELISA, Biotest AG, Dreieich, Germany); In addition, other techniques such as the Rose Bengal test and IgM/IgG flow assay for brucellosis [24], Heamaglutination Inhibition for Chikungunya, IFA (Panbio, Queensland, Australia) for rickettsisoses and a dipstick assay (KIT Biomedical Research, the Netherlands) for typhoid fever were also used. All the tests had been performed at the Institute of Virology, Eramus Medical Center, Rotterdam, the Netherlands and the Royal Tropical Institute, Amsterdam, the Netherlands and the department of Microbiology, Cho Ray hospital, Ho Chi Minh City, Vietnam. Details of the dengue ELISA have been described previously [25,26]. The sensitivity, specificity and the positive and negative predictive values of the combined IgM and IgG tests in paired sera were 85%, 94%, 65% and 98% for the diagnosis acute dengue, respectively [27].

In the pre-intervention period, we performed dengue ELISA for all eligible patients followed by testing for other diseases when acute dengue was ruled out. During the intervention period, these tests were done for two randomly chosen patients per health facility per month, complemented with all patients for whom a dengue RDT was used in 2005. Dengue ELISA results were classified into "acute dengue" (primary and secondary dengue) and "no acute dengue" (recent, past and no dengue), following a previously published algorithm [27].

### Interventions

After a pre-intervention period of one year, from April 2001 until April 2002, the participating PHCs were randomized into four intervention groups studying two interventions, *viz*. improvement of knowledge (intervention A), and the introduction of RDTs (intervention B), using a factorial design. Intervention A consisted of a two-day training course for PHC staff on the clinical signs and symptoms of the infectious diseases that are prevalent in Binh Thuan and introduction of a diagnostic algorithm that was adapted from existing algorithms and guides through a range of signs and symptoms to a presumptive diagnosis [28]. The results of serological tests were unveiled to the staff of participating PHCs to raise their awareness about the incidence of diseases. Intervention B consisted of the introduction of RDTs, to be used by health staff when considered indicated. The RDTs included tests for leptospirosis (Leptotek dridot ^®^) and typhoid fever (Royal Tropical Institute, Amsterdam, the Netherlands) [29,30]. For dengue we used the PanBio Dengue Duo Cassette test (PanBio, Queensland, Australia) based on previous comparisons to other tests [25,31]. This dengue test was only introduced in November 2004 due to production and delivery problems [32]. Participants were trained in the use of RDTs. The presumptive diagnosis made with or without the aid of RDTs was the outcome of this study, not the test result itself. Unclear readings of RDTs were recorded as indeterminate and interpreted conservatively but in principle left to the distinction of the health care worker.

The introduction of RDTs included training and providing equipment for conducting a full blood count (FBC). In the training course the health staff was equipped with knowledges about RDTs as well as practical skills that were transferred by experts from Cho Ray hospital and the Royal Tropical Institute, the Netherlands. However, several PHCs in Binh Thuan were already able to conduct FBCs, including some PHCs that were not randomized to receive RDTs (Table 1 and Figure 1).

### Outcomes

Measures of outcome included the presumptive and confirmed diagnoses, the treatment of febrile patients and costs. Where introduction of RDTs was part of the intervention, the presumptive diagnosis was made after deciding whether to use the RDT. If an RDT was used, the presumptive diagnosis was made after reading the RDT. For all patients the attending healthcare workers recorded data on self-treatment and costs of pre-referral treatment, presumptive diagnosis, RDT results (during the intervention period), prescribed treatment and costs paid by the patient to obtain the prescribed medication, whether referral was needed and final outcome. Presumptive diagnoses such as "acute fever" and "viral infection" were re-classified to "undifferentiated fever" (UF).

To explore the effect of interventions on diagnostic behaviour, two concepts were introduced: diagnostic resolution and accuracy. Diagnostic resolution was expressed in two ways; first the diagnostic resolution for UF: the frequencies of cause specific diagnoses as opposed to "UF", and second the diagnostic resolution for dengue: the frequency of the presumptive diagnosis "dengue" versus any other diagnosis not being dengue. Diagnostic accuracy was expressed as the predictive value of the presumptive diagnosis of dengue, i.e. the percentage that was serologically confirmed. Dengue was singled out for this as it was the most common cause of fever and thus key in avoiding unnecessary antibiotic use.

To explore the effect of interventions on medical costs two sources of health expenses, paid by the patients, were recorded: expenses for consultations or drugs before consulting the PHC (which should not be affected by our interventions) and costs for consulting the PHC and drugs prescribed there. Self-medication or drugs prescribed by another health care provider, taken before consulting the PHC, were elicited including the name of the drugs with their dosage, duration and costs in Vietnamese Dong (but here expressed in USD, taking a fixed exchange rate of 1USD = 15400VND - the exchange rate in 2003; http://www.vietcombank.com.vn/en/Exchange Rate.asp. The recently introduced health insurance caused confusion in recording expenses during the pre-intervention period and so these data were not used for analysis. During intervention costs for insured patients were calculated as if they were non-insured. Expenses for drugs were calculated using a list of standardised drug prices available at PHCs. Consultation fees were added to the total expenses. The costs of RDTs were not included in the health expenses.

### Statistical analysis

The analysis of discrete outcomes, specifically diagnostic resolution (i.e. is a causal diagnosis given or not) and accuracy (is diagnosis correct or not) was based on two approaches. First the pre-intervention period (as a whole) was compared to the intervention period (as a whole) within and between groups. Effects were expressed as odds ratios (OR). ORs were calculated using the Mantel-Haenszel method with each of the permanent health workers as strata. (Note: this essentially treats staff members as "fixed effects" and does not readily allow generalization to all health care workers. In view of the non-random selection of these health workers such generalization would have been unjustified anyway). Differences among health staff members were explored using a test for heterogeneity among strata.

Second, to detect changes over time during the intervention phase (perhaps due to learning effects), the effects of interventions and the interaction with the study period, were estimated using conditional logistic regression with time (in months) as continuous covariates.

For the continuous outcome "costs" to compare before and after intervention, within and among trial arms, the rank test for medians was used. In addition, the effects of the interventions were explored by univariate General Linear Models (GLM) for the fifteen permanent doctors, with time (in months) as a covariate and interventions as a fixed factor. Data analyses were conducted with SPSS (version 15.01, SPSS Inc., Illinois).

## Results

### Health staff and patients

A total 60 primary health staff members participated, who enrolled a total of 14512 patients. There were fifteen permanent health staff members, 8 female and 7 male, with an average age of 45.7 years (range 32-60 years) who participated throughout the entire study period. At the beginning of the study, in 2001, five of them had an MD degree and 10 were assistant doctors. In 2004, one of these graduated with a MD degree. Their mean (range) working experience was 23.2 (10 to 40) years.

The 15 permanent health staffs enrolled 7654 (52.7% of the total) patients, 1321 pre-intervention and 6333 during intervention. The median and 95th percentile number of days delay between symptom onset and presentation to the PHC were 1.5 and 3 days, respectively. Before consultation, 3037 (39.7%) of patients received some sort of medical help or self-treatment. This declined from 781 (59.1%) pre-intervention to 2256 (35.6%) during intervention.

Twenty-one different presumptive diagnoses were recorded during the entire study period. More than half of the patients (4162 - 54.4%) received the non-specific diagnosis "UF". Fewer patients received an etiologic diagnosis such as "dengue" (400 - 5.2%), "leptospirosis" (18 - 0.2%), "typhoid fever" (49 - 0.6%), "mumps" (6 - 0.1%), "measles" (2 - 0.0%), or "rickettsiosis" (1 - 0.0%). The majority of other diagnoses referred to an anatomical site of infection such as "pharyngitis" (2501 - 32.7%), "tonsillitis" (360 - 4.7%) or "diarrhoea" (97 - 1.3%) (Table 2). Usage of RDTs was surprisingly low (Table 3).

**Table 2:** Characteristics of the febrile patients consulting 15 permanent health staffs, their presumptive diagnosis and the antibiotic treatment.

	Pre-Intervention	Intervention					p-value^b)^
			
		Training	RDTs	Training + RDTs	Control	p- value^a)^	
**No. of patients included**	1321	1120	2798	1516	899		
**Median age **(years, range)	15.9 (1 - 82)	13.9 (1- 85)	21.4 (1 - 83)	15.4 (2 - 80)	15.1 (1 - 74)	< 0.001	0.024
Child/Adult ratio	1.0	1.4	0.5	1.0	1.0	< 0.001	0.009
**Sex **^c)^ F/M ratio	0.7	1.1	0.8	1.0	0.6	< 0.001	0.014
**Season **Dry/Rainy ratio	0.9	0.7	0.9	0.8	1.3	< 0.001	NS
**Occupations **(%)^d)^							
Farmer	33	20	54	35	35	< 0.001	< 0.001
Pupil	42	42	30	52	47		
Other	25	38	16	13	18		
**Presumptive Diagnoses (%)**						< 0.001	< 0.001
Acute Fever	56	67	52	53	45		
Pharyngitis	22	13	46	26	43		
Dengue	9	4	1	12	3		
Tonsillitis	6	11	0	7	6		
Other ^e)^	7	5	1	2	3		
**Antibiotic treatment (%)**	77	81	57	44	90	< 0.001	< 0.001
**Intravenous fluid (%)**	23	8	51	13	2	< 0.001	< 0.001

p-value (a): for intervention groups; p-value (b): for pre-intervention and intervention groups, p-value by chi-square test.

c): non specified: 7; d) non specified: 60; e) 12 other diagnoses: Diarrhoea, Typhoid fever, Leptospirosis, Hepatitis, Varicella, Allergy, Arthritis, Clinical Malaria, Gastritis, ARI, Lymphadenitis, Measles.

**Table 3:** The usage of rapid diagnostic tests

	By non-permanent staff, n (%)	By permanent staff, n (%)		
		
		Training	RDTs	Training + RDTs
Full blood count	1329 (27)	17 (3)	1111 (41)	371 (25)
Leptospirosis test	50 (2)		140 (5)	218 (14)
Typhoid test	6 (0)		2 (0)	22 (2)
Dengue test	124 (15)		83 (8)	55 (14)

All consultations were concluded with a prescription for drugs such as antipyretics (for 97.1% of patients), antibiotics (65.0%), vitamins or tonics (76.4%), corticosteroids (4.1%), parenteral fluid (26.7%) and others (43.1%). Antibiotics were prescribed for 96.4% of patients who had a presumptive diagnosis of "tonsillitis", for 95.6% of patients with "pharyngitis", for 45.0% of patients with "dengue", for 44.8% of patients with "UF" and for 82.7% of patients with other diagnoses. Parenteral fluid was administered to 30.0% patients with the presumptive diagnosis "dengue", but also to patients with "pharyngitis" (30.1%), "UF" (26.2%), "tonsillitis" (94.2%) and other diagnoses (27.3%).

### Effects of interventions

Diagnostic resolution for UF, expressed as the frequency of presumptive diagnoses being more specific than "UF", was lower during intervention than before in group AB only but this change did not reach statistical significance. The frequency of "UF" as a presumptive diagnosis increased in groups A (OR 2.102, 95%CI: 1.582-2.791, p-value: <0.001) and B (1.388, 95%CI: 1.003-1.785, p-value: 0.048) and also increased in group C, but not significantly (Table 4). There was significant heterogeneity among individual staff members, especially in group C, which may conceal differences between PHCs and interventions.

**Table 4:** The effects of interventions on diagnostic resolution, diagnostic accuracy of dengue and prescription of antibiotics

Interventions	Intervention effects, M-H Odds ratios of association between pre/post intervention and correctness of diagnosis (95% CI)			
	
	Diagnostic resolution		Diagnostic Accuracy of Dengue ^3)^	Prescription of Antibiotics
			
	"Undifferentiated fever"^1)^	"Dengue" ^2)^		
Training	2.1 (1.6-2.8)^a)^	0. 3 (0.2-0.5)^a)^	0.8 (0.3-2.0) ^c)^	1. 1 (0.7-1.5)^c)^
RDTs	1.3 (1.0-1.8) ^b)^	0.3 (0.1-0.6) ^a)d)^	1. 4 (0.8-2.4)^c)^	0. 7 (0.5-0.9)^a)^
Training + RDTs	0.9 (0.7-1.1)^c)^	1.5 (1.0-2.2)^c)^	1.9 (1.1-3. 5)^b)d)^	0.1 (0.0-0.1)^a)^
Control	1.2 (0.9-1.6)^c)d)^	0.3 (0.2-0.5) ^a)d)^	1.1 (0.4-3.2)^c)^	10.3 (6.9-15.4)^a)^

1) "undifferentiated fever" as a presumptive diagnosis, compared to all more specific diagnoses; 2) "dengue" as a presumptive diagnosis, compared to diagnoses not being dengue; 3) the serologically confirmed presumptive diagnosis of dengue or not dengue.

a) p-value: ≤ 0.002; b) 0.05 > p-value ≥ 0.01; c) p-value: ≥ 0.05 d) significant homogeneity among individual health staff members within the intervention group.

**Figure 2:**
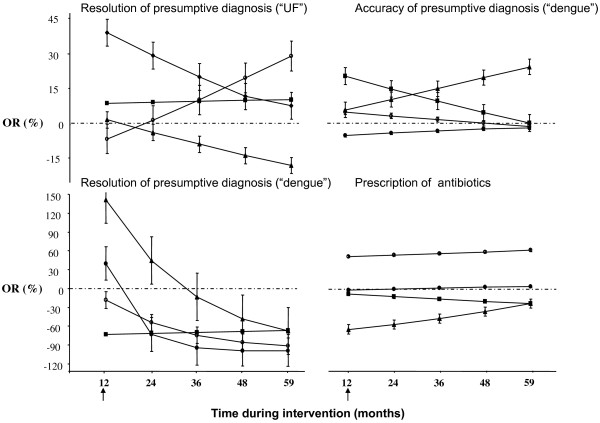
**Time dependent changes of the diagnostic resolution, diagnostic accuracy and prescription of antibiotics for undifferentiated fever during different interventions**. Diagnostic resolution for undifferentiated fever (UF) is expressed as the odds ratio (OR) for the non-specific diagnosis UF versus any specific presumptive diagnosis. Diagnostic resolution for dengue is expressed as the OR for a presumptive diagnosis dengue versus any other diagnosis not being dengue. Diagnostic accuracy is expressed as the OR for a correct presumptive clinical diagnosis on dengue (positive or negative), confirmed by serology, versus a false presumptive diagnosis. Prescription of antibiotics is expressed as the OR for prescribing antibiotics versus no such prescription. The different interventions groups are shown separately: A: solid circle; B: solid square; AB: solid triangle; C: empty circle. The arrow at the abscissa indicates the start of the intervention period.

During intervention phase, the trends of diagnostic resolution for UF (by conditional logistic regression) are shown in Figure 2. In the control group this showed a declining trend (p-value: 0.077). There was a significant increase of the proportion "UF" as presumptive diagnosis in group A, i.e. a decline in diagnostic resolution for UF, followed by a return to the pre-intervention level (p-value: 0.048). In group B there were no changes in diagnostic resolution for UF. For group AB there was a trend towards a diagnosis that was more specific than "UF" (p-value: 0.095).

Diagnostic resolution for dengue, expressed as a presumptive diagnosis "dengue" versus "no dengue" was higher during intervention, compared to pre-intervention, but insignificant in group AB (OR = 1.478, 95%CI: 1.000-2.184, p-value: 0.050). In other groups the frequency of "dengue" was significantly lower during intervention than before (group A: OR = 0.283, 95 CI%: 0.178-0.451, p-value: <0.001; group B: OR = 0.268, 95% CI: 0.120-0.598, p-value: 0.001; group C: OR = 0.301, 95% CI: 0.167-0.541, p-value: <0.001), with considerable heterogeneity only in group A. During intervention, the diagnostic resolution for dengue initially increased in group AB followed by a decline (p-value: <0.001). In groups A and C this declined significantly (p-values: <0.001 and 0.005 respectively). In group B the diagnosis "dengue" initially declined but remained stable during the rest of the intervention period (Figure 2).

Diagnostic accuracy for dengue was analysed by comparing the presumptive diagnosis to the ELISA results. Out of a total of 1929 patients who received a sero-diagnosis, 1067 patients (488 - 45.7% pre-intervention and 579 - 57.3% during intervention) were managed by the fifteen permanent health staffs, and of these 176 (16.5%) had confirmed acute dengue.

Table 5 presents sensitivities, specificities, positive predictive values (PPVs), and negative predictive values (NPVs) of the presumptive diagnosis "dengue" before and during intervention for the 4 trial arms.

**Table 5:** Sensitivity, specificity, positive predictive value (PPV) and negative predictive value (PNV) of the presumptive diagnosis "dengue" in pre-intervention/intervention period in the 4 trial arms

	Training		RDTs		Training + RDTs		Control	
	
	Pre-intervention	Intervention	Pre-intervention	Intervention	Pre-intervention	Intervention	Pre-intervention	Intervention
*Acute dengue among AUF patients (%)*	*27.3*	*18.6*	*18.8*	*15.0*	*28.6*	*21.4*	*20.5*	*12.5*

Sensitivity (%)	**23.1**	**0.0**	**3.2**	**3.3**	**12.1**	**30.0**	**0.0**	**0.0**
95% CI	0.2 - 46.0	-	0 - 9.4	0 - 9.8	3.7 - 20.5	1.6 - 58.4	-	-
	
Specificity (%)	**96.2**	**96.5**	**93.4**	**93.5**	**93.7**	**90.4**	**97.1**	**96.4**
95% CI	90.9 - 100	92.6 - 100	89.2 - 97.6	90.3 - 96.8	89.9 - 97.5	85.2 - 95.6	91.4 - 100	92.4 - 100
	
PPV (%)	**60.0**	**0**	**10.0**	**6.7**	**41.2**	**20.0**	**0**	**0**
95% CI	17.1 - 100	-	0 - 28.6	0 - 19.3	17.8 - 64.6	0 - 40.2	-	-
	
NPV (%)	**83.3**	**83.0**	**80.9**	**87.5**	**74.4**	**94.2**	**84.6**	**87.9**
95% CI	73.9 - 92.8	75.6 - 90.4	74.7 - 87.0	83.2 - 91.8	68.3 - 80.4	90.0 - 98.4	73.3 - 95.9	81.2 - 94.6

The diagnostic accuracy for dengue was higher during intervention, compared to pre-intervention, in group AB (OR = 1.948, 95%CI: 1.088-3.485, p-value: 0.025) and also in groups B and C, but not significantly. In group A, there was no difference. The trends of diagnostic accuracy for dengue over time during intervention are presented in Figure 2. There were some changes over time but none of these reached statistical significance.

There were some differences for drug prescription between the intervention groups. Overall, the health staffs in groups A and C prescribed antibiotics more frequently than in groups B and AB. In contrast, the health staffs in group B administered more intravenous fluid to their patients (Table 2).

In comparison to the pre-intervention period, prescriptions for antibiotics increased significantly during intervention in group C (OR = 10.334, 95%CI: 6.918-15.438, p-value: <0.001) and slightly, not significantly, in group A; it declined in groups B (OR = 0.657, 95%CI: 0.507-0.852, p-value: 0.002) and AB (OR = 0.106, 95%CI: 0.076-0.147, p-value: <0.001) (Table 4) However, there was considerable heterogeneity among the health staff members of all groups. The initial decline in group AB was followed by a slight but significant increase over time during intervention (p-value: <0.001). Surprisingly, the initial large increase of antibiotic prescription in the control group remained stable over time. In group B there was a trend towards a declining prescription of antibiotics during intervention (p-value: 0.049) (Figure 2).

### Health expenses

**Figure 3:**
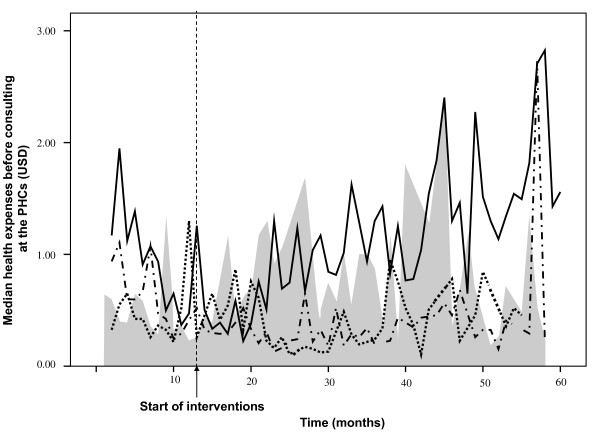
**Health expenses paid for undifferentiated fever before consulting the public primary health facility**. Median health expenses (USD) for intervention groups: A: dot line; B: solid line; AB: dash dot line; The control group (C) is depicted as the gray area.

The overall pre-consultation health expenses, including drugs and consultation costs, were all paid out of pocket and remained approximately constant, amounting to 0.60 USD (range: 0.03-29.22 USD) before intervention and 0.64 USD (range: 0.05-12.99 USD) during intervention (p-value: 0.299). Figure 3 presents the pre-consultation health expenses. Costs, overall, before and during intervention were in group A: 0.37 USD, 0.36 USD, 0.38 USD respectively (p-value: 0.424); B: 0.98 USD, 0.94 USD and 1.00 USD (p-value: 0.244); AB: 0.35USD, 0.58 USD, 0.30 USD (p-value: <0.001); and group C: 0.62 USD, 0.38 USD, 0.72 USD (p-value: <0.001; overall p-value: < 0.001).

**Figure 4:**
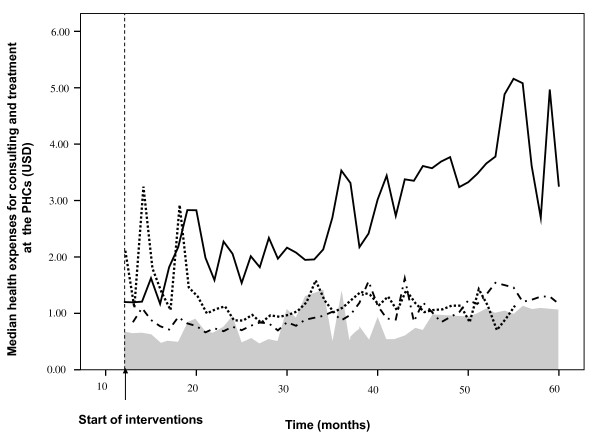
**Costs paid for medical care delivered for undifferentiated fever at public primary health facilities**. Median health expenses (USD) for intervention groups A: dot line; B: solid line; AB: dash dot line. The control group (C) is depicted as the gray area.

During intervention the median of the expenses made to purchase the prescribed drugs plus consultation fees was 1.43 USD (range: 0.06-38.96 USD). There were significant differences among the intervention groups. Median costs were, ranked from the highest to lowest, 3.11 USD (range: 0.06-38.96) in group B, 1.11 USD (range: 0.06-25.97) in group A, 0.95 USD (range: 0.06-7.79) in group AB and 0.92 USD (range: 0.13-4.55) in group C (p value: < 0.001, Rank test). Analysis with GLM showed similar results (data not given). Figure 4 presents the health expenses during the intervention period for each intervention group.

## Discussion

This study showed that the separate interventions, post graduate training on infectious diseases with (clinical) epidemiology and the introduction of RDTs is not enough to improve the diagnostic resolution and accuracy of the presumptive diagnosis for undifferentiated fevers, including dengue, or reduce the prescription of antibiotics or overall costs. Only the combined approach, training plus RDTs, improved diagnostic resolution and diagnostic accuracy for dengue, and reduced the prescription of antibiotics as well as expenses for medication.

This study is special by its exploring primary health care level interventions that target diagnostic and therapeutic behaviour of health professionals with respect to fevers in an area where malaria had been brought under control. Our study was carried out in an era of change and this, as well as mobility of health workers, affected effective sample size and outcomes. Both algorithms or guidelines, and adherence of physicians to these, are often deficient for various reasons [33,34]. In this study we did not measure adherence to guidelines and so cannot distinguish between poor guidelines (algorithms) or poor adherence to these guidelines as the cause of the poor quality of presumptive diagnoses.

The study tried to capture trends in diagnostic and therapeutic responses of health care workers, as they occur in real life. It shows that routine health care systems are rather inert and insensitive to change and that variability between and within health centers is considerable and perhaps requires larger sample sizes. The delayed introduction of the dengue rapid tests may have introduced some diminution of effects. Despite the rather aggressive marketing of rapid diagnostic tests, these were still new at the PHCs in Binh Thuan, where laboratory support was limited to for a full blood count and a blood smear for malaria. Improving the usage of RDTs itself was not the aim of this study, this was deliberately left to the considerations of the health care providers; we only provided the tests and the instructions for their use. Nevertheless, the tests were used erratically and less than expected. The most striking illustration is the very infrequent use of a full blood count at the PHC in group A, despite the availability of facilities. We did not study the motives for using the tests but most of the time the health staff members felt probably secure enough in their diagnostic and therapeutic practice without the assistance of a rapid test.

In this study the presumptive diagnosis "acute dengue" was not specified in primary or secondary dengue. Although complications occur more often in secondary dengue than in primary dengue, the vast majority of patients with secondary dengue do not develop complications. With antibody based tests the distinction cannot be made in the acute stage of disease, but the combination of a RDT based on NS1Ag in combination with an IgM/IgG tests is very promising and can perhaps assist in differentiating case management for primary and secondary dengue [35].

The prescription of antibiotics significantly declined in the group that received training as well as RDTs. This was also the only group in which the diagnostic accuracy increased significantly. However, the improvement was not sustained over time, consistent with finding from other studies [36,37]. Nevertheless, even in this group the positive predictive value of dengue presumptive diagnoses was still low. Perhaps even too low to rule out a bacterial etiology for patients with this diagnosis and discharge patients without follow up.

A cause for concern was the increase in costs over time, especially in group B. This should be considered against a background of only slightly increasing expenses for pre-consultation care and self-medication. Out of pocket payments for health may exacerbate poverty in poor countries [38]. In Vietnam, the impact on poverty by such payments is declining, but still in 1998 it caused an increase of the proportion of people below the food poverty line from 15% to 18,4% [39] With an incidence of large unexpected household spending for health calamities of more than 10%, Vietnam ranked among the highest 59 countries [40], although this incidence continuously declined over the period 1993-2006 [41].

Interestingly, we observed that the increase of costs in group B was not so much caused by prescription of drugs, but by administration of IV fluids, for which we have no explanation. This tendency to revert to more invasive treatments is difficult to explain. We don't have an explanation for this behaviour. It may have been elicited by the introduction of RDTs without evidence based guidance (since this was not part of the interventions) but should be studied further.

The very short patient delay in this study is remarkable. It points at a high consumption of health care and may drive casual diagnostic and treatment practices. In this study, febrile patients usually sought help at PHCs within 2 days from onset of fever [3], which often was the only sign of disease. Their presentation was often so non-specific that even the algorithms for making presumptive and differential diagnoses and using RDTs were of little use. This short patient delay, which contributes to such non-specific presentation, may be attributable to the health education component in the national and provincial malaria control programs which emphasized short patient delays [42]. However, the habit to seek prompt diagnosis and treatment is probably one of the driving forces to prescribe antibiotics for patients with self limiting viral infections. An inverse mechanism can be observed in the malaria holoendemic areas in Africa, where over-diagnosis of malaria results in over-treatment for malaria and missing other causes of fever [43].

## Conclusion

In conclusion, under conditions of growing health consumption and increasing costs for health services, the introduction of rapid diagnostic tests for infectious diseases such as dengue, through free market principles, does improve the quality of the diagnosis and decreases the prescription of antibiotics. However, the introduction of these tests may cause an increase of costs, not only by the price of the test itself but also by parallel mechanisms. The negative effects of the introduction of RDTs can be redressed by concomitantly providing education, but the effects of education are short lived. This is a plea for continuous postgraduate education and for a restriction for these tests to be used only by health professionals with an understanding of diagnostic procedures and guidelines.

## Competing interests

The authors declare that they have no competing interests.

## Authors' contributions

HLP collected the data, implemented the interventions, managed serum archive, carried out the immunoassays, performed the statistical analysis and drafted the manuscript. TTTN managed serum archive, carried out the immunoassays. PTG and LQH participated in it's design, collected the data and implemented the interventions. TQB participated in the design of the study, implemented the interventions and monitored overall the study. NVN participated in the design of the study, implemented the interventions and monitored the study at the field. NN participated in the design of the study, performed the statistical analysis and helped to draft the manuscript. PJdV participated in the design of the study, implemented the interventions, supervised overall the study, performed the statistical analysis and drafted the manuscript.

All authors read and approved the final manuscript.

## Pre-publication history

The pre-publication history for this paper can be accessed here:

http://www.biomedcentral.com/1472-6963/10/275/prepub

## Supplementary Material

Additional file 1 **CONSORT 2010 Flow Diagram**. this file contains number of patients who were enrolled in the study Click here for file
