# Supplementary material for: Randomised primary health center based interventions to improve the diagnosis and treatment of undifferentiated fever and dengue in Vietnam
Source: BMC Health Serv Res. 2010 Sep 21;10:275. doi: 10.1186/1472-6963-10-275 (PMC2955016; doi:10.1186/1472-6963-10-275)
Supplement: Additional file 1 — CONSORT 2010 Flow Diagram. this file contains number of patients who were enrolled in the study [file 1472-6963-10-275-S1.DOC]

**CONSORT 2010 Flow Diagram**

Excluded:

♦  Not meeting inclusion criteria 83 patients

**Assessed for eligibility**

**(13 PHCs, 60 staff and 14595 patients)**

**Pre-intervention, 1 year**

No. PHCs:

No. staff:

No. enrolled patients:

3

7

714

3

8

359

3

7

579

4

13

454

8

2280

378 1902

8

1184

51

1133

10

1468

183

1285

19

1926

173

1753

*Excluded from analysis:*

No. non-permanent staff:

No. enrolled patients:

pre-intervention:

intervention:

***Intervention:***

No. PHCs:

No. staff:

No. enrolled patients:

**Analysis**

**Intervention period , 4 years**

A

3

11

3022

AB

3

14

2801

B

3

12

3931

Control (C)

4

23

2652

3

3

1456

336

1120

3

4

3106

308

2798

3

4

1912

396

1516

4

4

1180

281

899

***Analysed:***

No. PHCs:

No. permanent staff:

No. enrolled patients:

pre-intervention:

intervention:
